# Mutation Spectrum of Stickler Syndrome Type I and Genotype-phenotype Analysis in East Asian Population: a systematic review

**DOI:** 10.1186/s12881-020-0963-z

**Published:** 2020-02-10

**Authors:** Dan-Dan Wang, Feng-Juan Gao, Fang-Yuan Hu, Sheng-Hai Zhang, Ping Xu, Ji-Hong Wu

**Affiliations:** 1 Eye Institute, Eye and ENT Hospital, College of Medicine, Fudan University, Shanghai, China; 2 Shanghai Key Laboratory of Visual Impairment and Restoration, Science and Technology Commission of Shanghai Municipality, Shanghai, China; 3 Key Laboratory of Myopia, Ministry of Health, Shanghai, China

**Keywords:** Stickler syndrome, *COL2A1*, Genotype-phenotype correlation, Retinal detachment, Gene therapy

## Abstract

**Background:**

Stickler syndrome is the most common genetic cause of rhegmatogenous retinal detachment (RRD) in children, and has a high risk of blindness. Type I (STL1) is the most common subtype, caused by *COL2A1* mutations. This study aims to analyze the mutation spectrum of *COL2A1* and further elucidate the genotype-phenotype relationships in the East Asian populations with STL1, which is poorly studied at present.

**Methods:**

By searching MEDLINE, Web of Science, CNKI, Wanfang Data, HGMD and Clinvar, all publications associated with STL1 were collected. Then, they were carefully screened to obtain all reported STL1-related variants in *COL2A1* and clinical features in East Asian patients with STL1.

**Results:**

There were 274 *COL2A1* variants identified in 999 patients with STL1 from 466 unrelated families, and more than half of them were truncation mutations. Of the 107 STL1 patients reported in the East Asian population, it was found that patients with truncation mutations had milder systemic phenotypes, whereas patients with splicing mutations had severer phenotypes. In addition, several recurrent variants (c.3106C > T, c.1833 + 1G > A, c.2710C > T and c.1693C > T) were found.

**Conclusions:**

Genotype-phenotype correlations should certainly be studied carefully, contributed to making personalized follow-up plans and predicting prognosis of this disorder. Genome editing holds great potential for treating inherited diseases caused by pathogenic mutations. In this study, several recurrent variants were found, providing potential candidate targets for genetic manipulation in the future.

## Background

Stickler syndrome is a clinically variable and genetically heterogeneous disease, first described by Stickler et al [1]. It is featured by ocular, skeletal, auditory and orofacial abnormalities. The incidence among newborns is approximately 1:7500–1:9000 [2]. Ocular findings include early-onset high myopia, retinal detachment (RD), cataract and glaucoma. It was reported that 50–73% of patients developed RD in their lifetime, and up to 75% of patients were bilateral [3–5]. Recurrent RD can cause irreversible vision loss and even blindness. Skeletal changes include joint hypermobility in childhood, mild spondyloepiphyseal dysplasia and premature osteoarthritis. Sensorineural hearing loss (HL) is usually mild, mainly for the high tones [6]. Orofacial features include flat midface, depressed nasal bridge, micrognathia and cleft palate.

At present, six subtypes of Stickler syndrome have been discerned. Type I (STL1, OMIM 108300) is the most common form, accounting for 80–90% [7]. STL1 is a dominantly inherited disorder, caused by mutations in the *COL2A1* gene (OMIM 120140). The *COL2A1* gene is mapped to human chromosome 12q13.11 and is composed of 54 exons, spanning 31.5 kb of genomic DNA [8]. It encodes α1(II) chain, which mainly expresses in hyaline cartilage, intervertebral discs, adult vitreous and inner ear [9].

To date, numerous patients with STL1 have been reported worldwide [9], but limited data are available in the East Asian population only with a few cases and small cohort studies. In this study, we aim to further elucidate the genotype-phenotype relationships by analyzing the clinical and genetic characteristics of East Asian patients with STL1. Furthermore, all reported variants in *COL2A1* associated with STL1 were also analyzed.

## Methods

MEDLINE, CNKI, Web of Science and Wanfang Data were searched, applying the following search terms for the period 1965 to October 2019: (mutation OR variant) AND (COL2A1 AND “Stickler syndrome”). In addition, Human Gene Mutation Database (HGMD) and Clinvar were also searched, and the search term was *COL2A1*. All relevant publications were carefully screened. We included publications that 1) were studies among patients with STL1, 2) provided the variant in *COL2A1*, and 3) were full-text articles. Reviews and obvious duplicates were excluded, and other types of studies met the inclusion criteria were included. Related information was collected, including variants and ethnicities. For patients in East Asia, detailed clinical features were also collected. All the processes were performed independently by two authors (D.W. and F.G.), including screening, selecting studies, extracting data and assessing the risk of bias. Any discrepancy in the assessment would be resolved by consensus. The study was performed according to the Declaration of Helsinki and approved by the Ethics Committee of the Eye and ENT Hospital of Fudan University.

All variants in *COL2A1* were checked to ensure that they were numbered according to the reference cDNA sequence NM_001844.4. If not, nucleotide and codon numbers were converted to ensure that their mutation nomenclature matched with reference transcript NM_001844.4 for *COL2A1*. For DNA numbering + 1 corresponded to the A of the ATG translation initiation codon. All statistical analyses were performed using SPSS 20.0 (SPSS Inc., Chicago, IL, USA). According to mutation types, East Asian patients were divided into 3 subgroups. Chi-squared test was performed to compare the phenotypes and sex among 3 subgroups. In addition, Kruskal-Wallis test was applied to compare the age among 3 subgroups. *P* value < 0.05 was considered the threshold of statistical significance.

## Results

### Spectrum of *COL2A1* Mutations

**Fig. 1:**
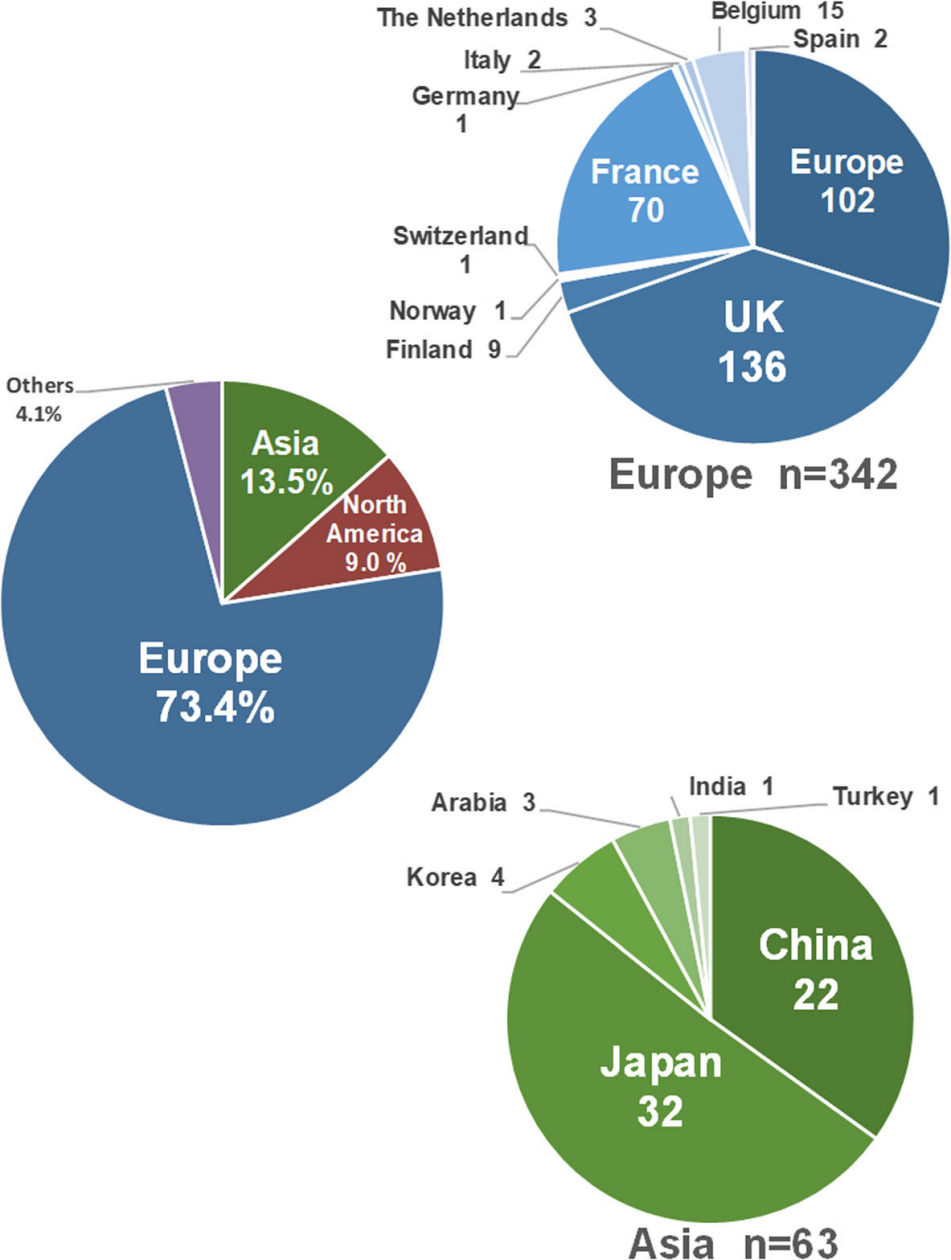
Population distribution of STL1-related *COL2A1* mutation reports. The pie-chart shows the number and percentage of STL1 patients harboring *COL2A1* variants from different ethnicities

**Fig. 2:**
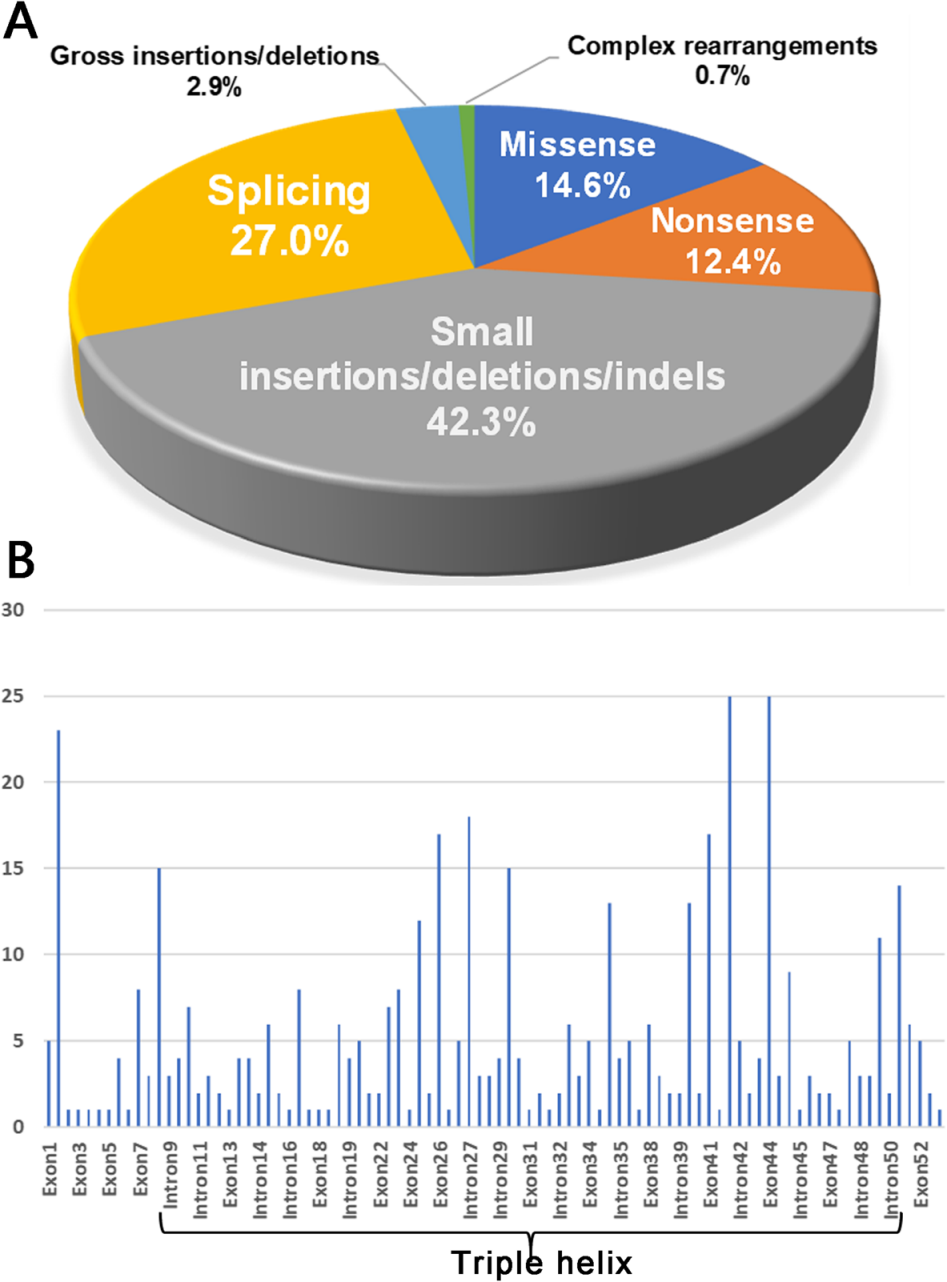
The distribution of variants in the *COL2A1* gene. **a** The distribution of mutation types. **b** The distribution of variants in different exon/introns

A total of 80 original articles met the inclusion criteria. There were 274 disease-causing variants in *COL2A1* identified in diverse ethnicities, which is shown in Additional file 1: Table S1. Of 999 patients with STL1 from 466 unrelated families, most were Europeans (342/466 families, 73.4%), followed by Asians (63/466 families, 13.5%) and North Americans (42/466 families, 9.0%; Fig. 1). All patients carried one heterozygous variant in *COL2A1*, except for one patient harboring two variants in *COL2A1*. Most variants (158/274, 57.7%) are nonsense and frameshift mutations (insertions/deletions/indels) that are predicted to cause premature protein truncation, leading to the absence of collagen synthesis (Fig. 2a). The majority of variants were identified in only one family (213/274, 77.7%), suggesting that the mutation spectrum is far from being saturated in spite of numerous *COL2A1* mutation reports.

### Mutation Locations

Several recurrent variants were identified in the European population, including c.3106C > T (15/342), c.1833 + 1G > A (12/342), c.1693C > T (10/342), c.2710C > T (9/342) and c.2353C > T (9/342). In addition, the variants c.1833 + 1G > A (5/58), c.1693C > T (4/58), c.3106C > T (3/58), c.2353C > T (3/58) and c.1957C > T were common in the East Asian population. Notably, the variant c.1833 + 1G > A was found in multiple ethnicities, but was not found in the North American population in spite of numerous STL1 reports (191 patients, 42 families). In addition, the variant c.258C > A was common in the North American population (4/42), and all 46 individuals carrying this particular allele were from the North America. It suggested that ethnicity contributes significantly to the distribution of mutations. No mutational hot region was found, but most variants in *COL2A1* were clustered in exon 42 (25/466, 5.4%), exon 44 (25/466, 5.4%) and exon 2 (23/466, 4.9%; Fig. 2b). To date, no disease-causing variant associated with STL1 has been identified in exons 20, 28, 53 and 54. Variants in these exons can cause severe phenotypes, such as spondyloepiphyseal dysplasia (SED) and spondyloepimetaphyseal dysplasia (SEMD).

The triple helix is the common structural feature of collagens, composed of a core repeat of Gly-X-Y [10]. The majority of variants in *COL2A1* were located in the triple helix region (encoded from codon 201–1214), accounting for 81.0% (222/274), followed by N-terminal propeptide (encoded from codon 26–181, 10.2%, 28/274). Corresponding to the enrichment of Gly-X-Y repeat, glycine replacement accounted for 22.6% (58/274), showing a predominant position. Variants located in other regions of type II collagen were rare.

### Clinical Features

**Fig. 3:**
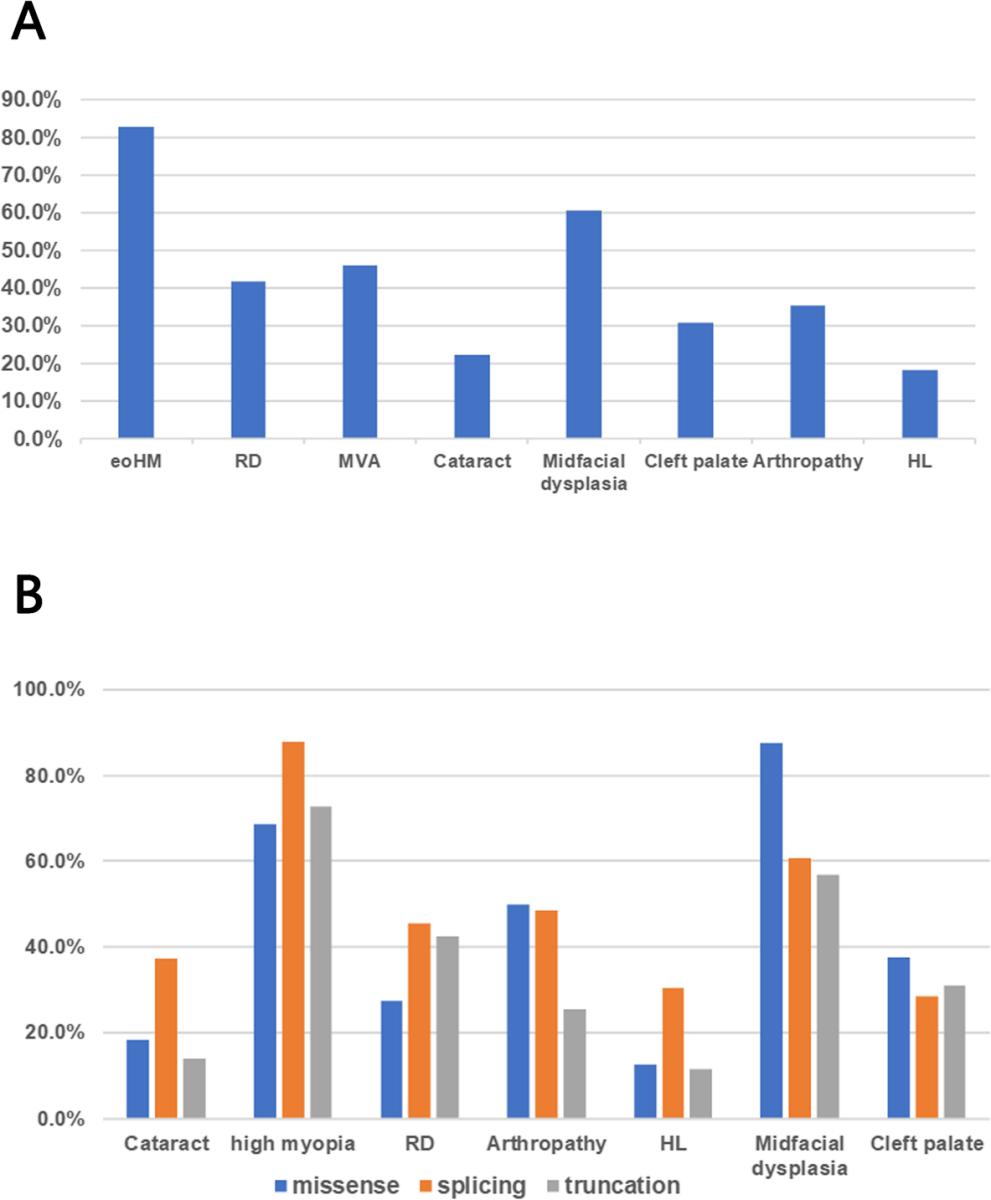
The clinical characteristics of 107 patients with STL1 in East Asia. **a** The prevalence of different phenotypes in STL1. **b** The prevalence of different phenotypes among 3 subgroups. eoHM: early-onset high myopia; RD: retinal detachment; MVA: membrane vitreous abnormalities; HL: hearing loss

A total of 107 STL1 patients (55 males, 52 females) harboring one heterozygous variant in *COL2A1* have been reported in the East Asian population. Thirty-two families from Japan, 22 from China, and four from Korea were included (Additional file 2: Table S2). The average age was 21.2 ± 16.2 years (range, 0.3–63.0; median, 15.0). All patients presented with ocular manifestations, and 82.8% (24/29) of eyes were high myopia before 6 years old. RD occurred in 41.7% (43/103) of the patients, and 46.5% (20/43) were bilateral. Membrane vitreous abnormalities (MVA) is a characteristic finding in patients with STL1 [10], observed in 45.8% (44/96) of the patients. In addition, 22.1% (23/104) of the patients had cataract, and only one patient had glaucoma. Skeletal changes occurred in 35.4% (34/96) of the patients, including precocious osteoarthrosis and joint hypermobility. Midfacial dysplasia, such as flat midface and depressed nasal bridge, is also a common manifestation, observed in 60.6% (60/99) of the patients, and 31.1% (19/61) of the patients presented with micrognathia. In addition, 30.7% (31/101) of the patients had cleft palate, 2 of which were submucous cleft palate. Sensorineural hearing loss was found in 18.1% (17/94) of the patients (Fig. 3a).

### Genotype-Phenotype Correlations

All East Asian patients were divided into 3 subgroups according to mutation types (Table 1, Fig. 3b). No apparent difference in age or sex was observed among 3 subgroups (*P* = 0.301 and 0.692, respectively). Compared with the other two groups, patients harboring splicing mutations were more likely to develop cataract (*P* = 0.004) and had severer systemic phenotypes (*P* = 0.010 in arthropathy, *P* = 0.022 in HL), whereas patients with truncation mutations had milder systemic phenotypes. In addition, there was no significant difference in the occurrence of high myopia, RD, midfacial dysplasia and cleft palate among 3 subgroups (*P* = 0.510, 0.575, 0.246 and 0.743, respectively).

**Table 1:** The Difference of Phenotypes in Patients with Different Mutation Types

Mutation types	Missense	Splicing	Truncation	*P* value
Cases	12	35	60	
Male/Female	5/7	20/15	30/30	0.692
Age, years	16.0 ± 13.8 (1.0–45.0)	23.2 ± 15.2 (2.3–62.0)	21.0 ± 17.1 (0.3–63.0)	0.301
Cataract	18.2%	37.1%	13.8%	0.004*
High myopia	68.8%	87.8%	72.8%	0.510
RD	27.3%	45.5%	42.4%	0.575
Arthropathy	50.0%	48.5%	25.5%	0.010*
HL	12.5%	30.3%	11.3%	0.022*
Midfacial dysplasia	87.5%	60.6%	56.9%	0.246
Cleft palate	37.5%	28.6%	31.0%	0.743

*RD* retinal detachment; HL: hearing loss; *: *P* < 0.05

Type II collagen can be alternatively spliced, and its long form including exon 2 mainly expresses in vitreous [11]. Therefore, patients with exon 2 mutations have few extraocular manifestations [12]. Nevertheless, seven patients harboring other exon mutations also had no systemic manifestation. In addition, phenotypic variation, such as differences in spherical equivalent refraction and sensorineural hearing loss or presence versus absence of cleft palate, and varying skeletal manifestations, was observed in siblings or in unrelated families with the same variants. This suggested the high clinical heterogeneity in this disorder, and there were other factors affecting the severity of the phenotypic spectrum, such as modifier genes and environmental exposures.

### Diagnostic Relevance

Of 107 patients with STL1, 10 patients were initially diagnosed with early-onset high myopia (eoHM), and diagnosis was not made correctly until they presented with systemic symptoms of STL1 after years of follow-up [13]. There were seven patients still diagnosed with eoHM, even if their *COL2A1* variants have been recorded in HGMD as STL1-related variants (Additional file 3: Table S3) [14, 15], because the diagnosis of STL1 is clinically based at present [16]. In addition, nine other patients with eoHM, harboring *COL2A1* variants, have been reported, and these variants have been recorded in HGMD as eoHM-related variants (Additional file 3: Table S3) [14, 15].

## Discussion

In this study, it was found that patients with splicing mutations had severer systemic phenotypes when compared with patients harboring truncation mutations and missense mutations. In addition, most STL1 patients carried with truncation mutations, and they were less likely to develop arthropathy. In light of the previous study, the STL1 spectrum was mostly attributed to truncation mutations, SED to missense mutations and Kniest dysplasia (KND) to splicing mutations [9, 17], which is consistent with our finding. This also explains why patients with STL1-related variants have milder phenotypes, especially arthropathy, than patients harboring other variants in *COL2A1*. Furthermore, this genotype-phenotype correlation provides a reference for personalized follow-up plans in spite of high clinical heterogeneity in STL1.

Genome editing shows great potential for treating inherited diseases caused by pathogenic mutations. Currently, multiple Cas9-based clinical trials are in progress or beginning soon, which is likely to guide future use for somatic cell editing both ex vivo and in patients [18]. Several recurrent variants were found in this study, including the variants c.3106C > T (18/466), c.1833 + 1G > A (18/466), c.2710C > T (15/466) and c.1693C > T (14/466), providing potential candidate targets for genetic manipulation in the future.

We reviewed clinical features of 107 STL1 patients in East Asia. There were 98.9% (88/89) of the patients with myopia, higher than that in the European population (89%) [19]. In addition, 82.8% (24/29) of eyes were high myopia before 6 years old. Zhou L et al. proposed that eoHM was the most easily recognizable sign for children with potential STL, usually presenting earliest [13]. Midfacial dysplasia was observed in 60.6% (60/99) of the patients, second only to myopia. However, it is also a common facial feature in Asians, which is easily ignored by clinicians. Therefore, some patients with STL1 are initially diagnosed with eoHM. Most phenotypes of STL1 become more prevalent with advancing age, such as RD, sensorineural hearing loss and arthropathy [20]. So, it is not wise to draw a conclusion too early, and a regular follow-up for multisystem is essential for patients harboring *COL2A1* variants.

STL is the most common genetic cause of rhegmatogenous retinal detachment (RRD) in children [21]. In this study, RRD was observed in 41.7% (43/103) of the patients and 46.5% (20/43) were bilateral, which is lower than previous studies (50%~ 73 and 75%, respectively) [3–5]. This may be related to the younger age of the patients included, because the prevalence of RRD is a function of age [20]. In addition, the prevalence of MVA was 45.8% (44/96), similar to the previous study in the European population (42%) [19]. There are some limitations in this study. The phenotypic data in some articles is incomplete, which limits the number of evaluable patients. Furthermore, the reporting bias should be inherent. The information based on the number of reports cannot reflect the prevalence of STL1 in East Asia. Lastly, a long-term, controlled prospective study is required to substantiate the genotype-phenotype correlation found in this study.

## Conclusions

In this study, our findings revealed that patients with splicing mutations had severer systemic phenotypes when compared with patients harboring other types of mutations, whereas patients with truncation mutations had milder phenotypes. This helps clinicians develop personalized follow-up plans for patients with STL1. In addition, recurrent variants c.3106C > T, c.1833 + 1G > A, c.2710C > T and c.1693C > T were found, which provides potential candidate targets for future gene therapy. Finally, high myopia before 6 years old is a key sign. For patients with high myopia, a regular follow-up for multisystem is essential if a heterozygous variant in *COL2A1* is identified.

## Supplementary information

**Additional file 1 : Table S1.** Variants of *COL2A1* in STL1 patients.

**Additional file 2 : Table S2.** Clinical characteristics in East Asian patients.

**Additional file 3 : Table S3.** The patients suspected with STL1.

## Data Availability

The datasets used and analyzed during the current study are available from the corresponding author on reasonable request.

## Abbreviations

eoHM: early-onset high myopia
HGMD: Human gene mutation database
HL: Hearing loss
KND: Kniest dysplasia
MVA: Membrane vitreous abnormalities
RRD: Rhegmatogenous retinal detachment
SED: Spondyloepiphyseal dysplasia
SEMD: Spondyloepimetaphyseal dysplasia
STL1: Stickler syndrome type I
